# *KRAS* mutation in lung metastases from colorectal cancer: prognostic implications

**DOI:** 10.1002/cam4.592

**Published:** 2015-12-29

**Authors:** Michele Ghidini, Nicola Personeni, Silvia Bozzarelli, Marina Baretti, Gianluca Basso, Paolo Bianchi, Maria Chiara Tronconi, Tiziana Pressiani, Fabio Grizzi, Laura Giordano, Alberto Malesci, Marco Alloisio, Luigi Laghi, Armando Santoro, Lorenza Rimassa

**Affiliations:** ^1^ Humanitas Cancer CenterHumanitas Clinical and Research CenterVia Manzoni 56, Rozzano20089MilanItaly; ^2^ Department of Medical Biotechnology and Translational MedicineUniversity of MilanVia Vanvitelli 3220129MilanItaly; ^3^ Laboratory of Molecular GastroenterologyHumanitas Clinical and Research CenterVia Manzoni 56, Rozzano20089MilanItaly; ^4^ Department of Inflammation and ImmunologyHumanitas Clinical and Research CenterVia Manzoni 56, Rozzano20089MilanItaly; ^5^ Department of GastroenterologyHumanitas Clinical and Research CenterVia Manzoni 56, Rozzano20089MilanItaly; ^6^ Humanitas UniversityVia Manzoni 56, Rozzano20089MilanItaly

**Keywords:** *BRAF*, brain metastases, cancer, colorectal, *KRAS*, lung metastases

## Abstract

*KRAS* mutant colorectal cancer (CRC) patients develop lung and brain metastases more frequently than *KRAS* wild‐type (WT) counterpart. We retrospectively investigated the prognostic role of *KRAS*,*BRAF*, and *PIK3CA* (exon 20) mutations and loss of phosphatase and tensin homolog (PTEN) in surgically resected lung metastases. Lung specimens from 75 metastatic CRC (mCRC) patients treated with one or more metastasectomies with curative intent were analyzed. Sixty‐four percent of patients had *KRAS* WT lung metastases. PTEN loss‐of‐function was found in 75%. *BRAF* and *PIK3CA* exon 20 mutations were not found. Seven patients subsequently developed brain metastases and 43% of them had *KRAS* mutation. In univariate analysis, median overall survival (OS) for *KRAS* WT patients was longer, compared to *KRAS* mutant patients (median 60.9 vs. 36.6 months, *P* = 0.035). In addition, both progression‐free survival (PFS) and lung disease‐free survival (LDFS) between lung surgery and relapse were not associated with *KRAS* and PTEN status. In multivariate analysis, the risk of death was significantly increased by *KRAS* mutational status (OS Hazard ratio (HR) 2.17, 95% IC 1.19–3.96, *P* = 0.012) and lack of adjuvant chemotherapy (OS HR 0.10, 95% IC 0.01–0.74, *P* = 0.024). The proportion of *KRAS* mutations in lung metastases was similar to the expected proportion in primary tumors. Patients harboring *KRAS* mutation had a poorer survival rate compared to WT group both in univariate and multivariate analysis. Moreover, administration of adjuvant chemotherapy after lung metastasectomy (LM) significantly improved both PFS and OS. *KRAS* mutation is a negative prognostic factor in mCRC patients undergoing LM. Further larger and prospective studies are necessary to confirm these findings.

## Introduction

Colorectal cancer (CRC) usually metastasizes to the liver (almost half of patients undergoing primary CRC resection will develop metachronous liver metastases and a quarter of patients diagnosed with CRC have synchronous hepatic secondaries) 1, 2. The lung is the most common extrahepatic site of metastases accounting for a 10–20% metastatization rate 2, 3. Lung recurrence occurs in 5–10% of patients who undergo surgery for localized CRC 4. Rectal cancer has a higher incidence of both synchronous (2.8‐fold increase in 5 years) and metachronous (2.63‐fold increase) pulmonary metastasization compared to colon cancer 3.

Several clinical factors, including a short disease‐free interval between the diagnosis of primary tumor and onset of lung metastases, multiple lung metastases (two or more), mediastinal and hilar lymph node involvement and elevated prethoracotomy serum carcinoembryonic antigen (CEA) levels, have been associated with reduced survival after pulmonary metastasectomy in patients with CRC 5.

Lung metastasectomy (LM) has become a widely accepted and safe procedure in the management of metastatic CRC (mCRC). Indeed, surgical practice has improved results obtained with stage IV palliative chemotherapy by increasing the 5‐year survival rate to more than 50% of patients with isolated pulmonary metastases, with an attested operative mortality of <1% 6, 7.

Despite the presence of clinical prognostic factors, none of the known molecular biomarkers has been clearly correlated with the prognosis of mCRC with lung metastases. Recently, it has been reported that patients with *KRAS* mutant CRC more frequently develop lung 8, 9, 10, 11, 12, 13, 14, 15 and brain metastases 9, 11. *KRAS* mutational status has been reported as a negative prognostic factor in many studies in early stage and mCRC 16, 17, 18, 19. Several reports are available on the negative prognostic role of both *KRAS* and *BRAF* mutation in patients undergoing liver resection 20, 21, 22.

Few series have focused on the negative prognostic role of *KRAS* mutation in the subset of patients with lung metastases 8, 9, 10, 11, 12, 13, 14, 15 and a recent series identified *BRAF* mutation as a significant negative prognostic factor as well 12. On the other hand, *PI3KCA* mutations were not found to have any prognostic implication in this selected cohort of patients 9, 11 while the role of phosphatase and tensin homolog (PTEN) loss has not been evaluated yet.

Here, we investigate the incidence and prognostic role of a panel of molecular biomarkers such as *KRAS, BRAF*, and *PIK3CA* (exon 20) mutations and loss of PTEN in a cohort of patients with mCRC undergoing LM.

## Material and Methods

We retrospectively reviewed the medical records of all patients treated with surgery for lung metastases from CRC at Humanitas Cancer Center, Rozzano, Milan, Italy, between 1997 and 2009. The study was approved by the Institutional Review Board. Patients were included in the analysis if (1) they had had a diagnosis of CRC (2) they had suffered from the development of synchronous or metachronous lung metastases (3) they had undergone one or more lung metastasectomies (4) pulmonary resection had been performed with a curative intent (5) tissue specimen of the pulmonary resection documented a diagnosis of mCRC and was available for molecular analyses. Lung metastases diagnosed within 6 months of the initial diagnosis of CRC were considered as synchronous 23. Both adjuvant chemotherapy for patients developing metachronous metastases and first‐line treatment for synchronous lung lesions were considered. For all patients fulfilling the inclusion criteria, we collected the following clinical characteristics: sex, date of birth and age, date of diagnosis and site of primary tumor, pathological tumor‐node‐metastasis and stage, date of diagnosis and sites of metastatic disease, number and site of lung lesions (left, right, unilateral or bilateral), number and type of systemic lines prior to lung surgery, type of adjuvant therapy, disease status before lung surgery (partial response, stable disease, progressive disease), date of lung surgery, outcome after surgery (relapse–nonrelapse), date of relapse, number and type of systemic lines of treatment after surgery, and date of last contact or death. We did not consider prethoracotomy serum CEA levels firstly because of the scarce reproducibility of dosages obtained in different laboratories and secondly because CEA elevation can be lacking in the setting of metastatic CRC to lungs. Indeed, prior studies have suggested that only 15% of patients with solitary lung metastases have a CEA elevation 24.

We evaluated the clinical outcome with respect to *KRAS, BRAF,* and *PIK3CA* exon 20 mutational status and loss of PTEN function in lung metastases.

**Figure 1:**
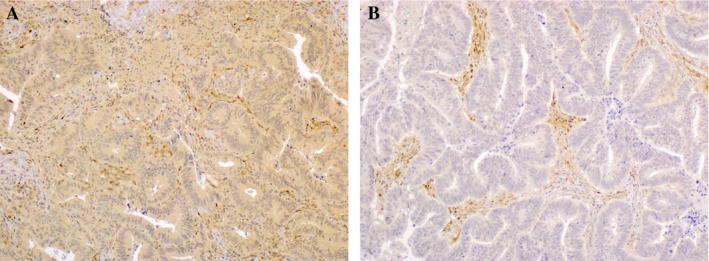
Phosphatase and Tensin Homolog immunohistochemistry (objective magnification 10 × ) (A) positive expression (B) negative expression.

PTEN expression was assessed by immunohistochemistry (IHC) using a monoclonal antibody (clone 6H2.1, 1:200; BioCare Medical, Concord, CA, USA), on 3 *μ*m thick tissues section. Results were expressed using a binary scoring system: positive PTEN expression was defined as staining in more than 10% of tumor cells, as previously reported (Fig. 1) 25.

*KRAS* (codon 12, 13, and 61) and *PIK3CA* exon 20 mutations were assessed in DNA extracted from paraffin‐embedded sections by direct sequencing. Each exon was amplified and sequenced. PCRs were performed in 50 *μ*L volumes containing 100 ng genomic DNA, 1× PCR buffer, 1.5 mmol/L MgCl2, 0.2 mmol/L each dATP, dCTP and dTTP, 0.2 *μ*mol/L each primer, and 0.5 units of Taq polymerase (Genespin, Milan, Italy). PCR products were purified with ExoSap‐it (USB^®^ Products; Affymetrics Inc., Santa Clara, CA, USA) following the manufacturer's instructions. Finally, 10 *μ*L of purified single‐strand DNA was submitted to sequencing analysis on the ABI PRISM 310 Genetic Analyzer (Applied Biosystems, Foster City, CA, USA). Each sequence was carried out at least twice, starting from an independent amplification reaction. The oligonucleotide primers used for amplifying the *KRAS* codon 12 and 13 were 5′‐TTATTATAAGGCCTGCTGAAAATG‐3′ (sense) and 5′CCTCTATTGTTGGATCATATTCGT‐3′ (antisense); for *KRAS* codon 61 were 5′‐GGAAGCAAGTAGTAATTGATGGAG‐3′ (sense) and 5′‐TTTATGGCAAATACACAAAGAAAG‐3′ (antisense). *PI3KCA* exon 20 was analyzed with 2 overlapping fragments (A and B): forward A primer was 5′‐ TCATTTGCTCCAAACTGACC‐3′ while reverse A primer was 5′‐ ACTCCAAAGCCTCTTGCTCA‐3′; forward B primer was 5′‐ CTCAATGATGCTTGGCTCTG‐3′ and reverse B primer 5′‐ TGGAATCCAGAGTGAGCTTTC‐3′.BRAF ^V600E^ mutation was determined by Real‐Time PCR using a TaqMan SNP Genotyping Assay (Applied Biosystem) on DNA extracted from paraffin‐embedded sections. TaqMan MGB probes were designed using the Custom TaqMan Assay Design Tool (Applied Biosystem). The chosen reporter fluorophores were VIC for detecting the wild‐type (WT) allele and FAM for the mutant allele 26.

### Statistical analysis

Differences in the distribution of demographics and clinic‐pathological characteristics between the molecular groups of interest were evaluated using the Chi‐square test, or the Fisher's exact test, as appropriate. Overall survival (OS) was calculated from the time of first pulmonary surgery to death (if alive, patients were censored at the time of the last contact). Progression‐free survival (PFS) was calculated from the time of first LM as well. Lung disease‐free survival (LDFS) was calculated between the date of surgery of primary tumor and lung relapse 23.

Actuarial survival curves were generated using the Kaplan–Meier method and differences between groups were estimated using the log‐rank test. A multivariable model was built to correct for the effect of confounders. Hazard ratio (HR) with its corresponding 95% confidence interval was calculated using the Cox Proportional Hazard Model. *P*‐value for statistical significance was set at <0.05. All the analyses were performed using R‐software (R foundation for statistical Computing, Wien, Austria).

## Results

### Patient characteristics

The main patients' characteristics are reported in Table 1. Seventy‐five patients were included. Median age at diagnosis was 65.4 years (range 33.4–80.1). Sixty patients (80%) had extrapulmonary disease of which 28 (37%) had liver metastases. Liver metastasectomy was performed in 25 cases. Only in two cases, a combined hepatic and pulmonary surgery was performed. Twenty (28%) patients had synchronous lung metastases while 53 (72%) developed metachronous pulmonary disease. In two cases (2%), time of development of lung metastases was not known. Twenty‐one patients (28%) underwent more than one surgery for metastases. Median number of lung metastases was 1 (range 1–10). Thirty patients (40%) developed right‐sided metastases only while 30 cases (40%) had left lung metastases only. In 15 cases (20%), both right and left‐sided metastases were diagnosed. All LMs were R0 or R1. Forty patients (54%) received chemotherapy prior to lung surgery while 35 patients (46%) did not receive systemic treatment. Among patients treated with preoperative chemotherapy, 14 patients (35%) underwent lung resection after the stabilization of disease or a partial response, while the majority (26 patients, 65%) underwent surgery after progressive lung disease. Twenty‐six patients (65%) received preoperative treatment either with FOLFOX or FOLFIRI, while the remaining had 5‐fluorouracil and folinic acid or other single‐agent chemotherapies. Eight patients (10%) had adjuvant treatment either with FOLFOX regimen or 5‐fluorouracil and folinic acid. Among these patients, 6 (75%) had primary surgery while 2 (25%) received also preoperative treatments. Fifty‐four patients (72%) relapsed after lung surgery and 41 of them (76%) had a subsequent systemic treatment. In 21 cases, *KRAS* mutational status was evaluated during the clinical course of the disease, and for the remaining 27 the mutational analysis was performed retrospectively. In nine WT patients, an anti‐epidermal growth factor receptor (*EGFR*) antibody (cetuximab or panitumumab) was administered while two patients with *KRAS* WT status received pulmonary stereotactic radiosurgery after relapse. Seven patients (9%) developed brain metastases and 43% of them had *KRAS* mutation.

**Table 1:** Baseline patient characteristics

Characteristics	Number (%)
Sex	
Female	48 (64)
Male	27 (26)
Age	
Median, years (range)	65.4 (33.4–80.1)
Primary tumor	
Colon	45 (60)
Rectum	30 (40)
Lung metastases	
Synchronous	20 (27)
Metachronous	53 (71)
Unknown	2 (2)
Localization	
Unilateral	60 (80)
Bilateral	15 (20)
Chemotherapy prior to lung surgery	
Yes	40 (53)
No	35 (47)
Adjuvant chemotherapy	
No	62 (89)
Yes	8 (11)
Extrapulmonary metastases	
Yes	60 (80)
No	15 (20)
Nodal involvement	
Yes	45 (60)
No	22 (29)
Unknown	8 (11)

Median follow‐up was 82.9 months (range 0.4–180.5). At the time of data collection, 48 (64%) of 75 patients had died. Median PFS was 13.1 months, median LDFS was 32 months while median OS 44.6 months.

### *KRAS*, PTEN, *BRAF*, and *PIK3CA* exon 20 analysis

*KRAS* mutations (exons 12, 13, and 61) were found in 26 cases (36%) while 48 cases were WT. Nineteen patients (25%) had intact PTEN while the majority (56 patients, 75%) had loss of protein expression. We did not detect any *BRAF* and *PIK3CA* exon 20 mutations, while exon 9 mutations were not investigated because effects on prognosis have been restricted only to exon 20 mutations 27.

The relationship between patient characteristics, *KRAS* mutations, and PTEN expression is shown in Table 2. Neither *KRAS* nor PTEN status were significantly associated with sex and location of primary tumor (colon or rectum). Furthermore, no associations were found between *KRAS* and PTEN status and time of onset of lung metastases (synchronous/metachronous), localization (unilateral or bilateral), presence or absence of extrapulmonary metastases, chemotherapy administration prior to or after lung surgery and nodal involvement. *BRAF* and *PIK3CA* exon 20 mutations were not detected.

**Table 2:** *KRAS* and PTEN analysis according to clinical factors on evaluable patients

Characteristics	*KRAS* WT*N* (%)	*KRAS* MUT*N* (%)	*P‐*value*KRAS* status	PTEN negative*N* (%)	PTEN positive*N* (%)	*P*‐valuePTEN status
All	48 (64)	27 (36)		56 (75)	19 (25)	
Sex						
Female	17 (63)	10 (37)	1.000	17 (63)	10 (37)	0.141
Male	31 (65)	17 (35)		39 (81)	9 (19)	
Primary tumor						
Colon	32 (71)	13 (29)	0.185	34 (76)	11 (24)	1.000
Rectum	16 (53)	14 (47)		22 (73)	8 (27)	
Lung metastases						
Synchronous	12 (67)	6 (33)	1.000	15 (83)	3 (17)	0.368
Metachronous	35 (64)	20 (36)		39 (71)	16 (29)	
Localization						
Unilateral	39 (65)	21 (35)	0.718	45 (75)	15 (25)	1.000
Bilateral	9 (60)	6 (40)		11 (73)	4 (27)	
Chemotherapy prior to lung surgery						
Yes	22 (55)	18 (45)	0.135	34 (85)	6 (15)	0.053
No	26 (74)	9 (26)		22 (63)	13 (37)	
Adjuvant chemotherapy						
No	41 (66)	21 (34)	1.000	45 (73)	17 (27)	1.000
Yes	5 (62)	3 (38)		6 (75)	2 (25)	
Extrapulmonary metastases						
Yes	41 (68)	19 (32)	0.207	44 (73)	16 (27)	0.745
No	7 (47)	8 (53)		12 (80)	3 (20)	
Nodal involvement						
Yes	27 (60)	18 (40)	0.308	33 (73)	12 (27)	0.550
No	16 (72)	6 (28%)		18 (82)	4 (18)	

PTEN, Phosphatase and Tensin Homolog; WT, wild‐type; MUT, mutant.

### Survival analysis

With a median follow‐up of 82.9 months, we observed a median PFS of 13.1 months and a median OS of 44.6 months. At the univariate analysis, PFS (median 11.0 months for pretreated patients, 21.4 months for nontreated, *P* = 0.040) and OS (median 28.4 months for pretreated, 73.3 months for nontreated, *P* = 0.005) were significantly shorter for patients who had chemotherapy prior to lung surgery. Moreover, patients who received adjuvant treatment showed longer PFS (median not reached for adjuvant treatment, 11.2 months for nonadjuvant treatment, *P* < 0.001) and OS (median not reached for adjuvant treatment, 42.8 months for nonadjuvant treatment, *P* = 0.010). An advantage in PFS (median 13.1 vs. 11.6 months, *P* = 0.026) and OS (median 58.0 vs. 28.5 months, *P* = 0.039) was also shown in patients with unilateral distribution of lung disease compared to bilateral disease (Table 3). Moreover, OS was significantly linked to *KRAS* mutational status (median 60.9 months for WT patients, 36.6 months for mutant, *P* = 0.035) (Table 4).

**Table 3:** Survival analysis according to baseline and clinical factors on evaluable patients

Characteristics	Median PFS (months)	*P*‐value PFS	Median OS (months)	*P*‐value OS
All	13.1		44.6	
Sex				
Female	13.4	0.081	58.0	0.265
Male	11.2		42.6	
Primary tumor				
Colon	13.2	0.308	43.2	0.647
Rectum	11.6		44.6	
Lung metastases				
Synchronous	13.2	0.542	43.2	0.607
Metachronous	13.1		51.1	
Localization				
Unilateral	13.1	0.026	58.0	0.039
Bilateral	11.6		28.5	
Chemotherapy prior to lung surgery				
Yes	11.0	0.040	28.4	0.005
No	21.4		73.3	
Adjuvant chemotherapy				
No	11.2	<0.001	42.8	0.010
Yes	NR		NR	
Extrapulmonary metastases				
Yes	13.1	0.849	43.8	0.916
No	10.7		51.1	
Nodal involvement				
Yes	13.1	0.824	42.4	0.170
No	11.7		60.9	

PFS, progression‐free survival; OS, overall survival; NR, not reached.

**Table 4:** Median PFS and OS according to *KRAS* and PTEN status

Characteristic	Median PFS months	*P*‐value	Median OS months	*P*‐value
KRAS				
Mutant	13.1	0.483	36.6	0.035
Wild‐type	13.1		60.9	
PTEN				
Positive	14.7	0.832	73.3	0.389
Negative	13.1		42.8	

PTEN, Phosphatase and Tensin Homolog; PFS, progression‐free survival; OS, overall survival.

PFS had no statistically significant association with both *KRAS* and PTEN status (Table 4). Moreover, there was no difference in the status of *KRAS* (median 33.1 WT vs. 32.0 months mutant, *P* = 0.402), and PTEN (median 30.8 negative vs. 39.1 months positive, *P* = 0.102) in determining LDFS between surgery of the primary tumor and lung relapse.

A multivariable model was built to correct for the effect of confounders statistically significant in the univariate evaluation. Disease localization and presurgical chemotherapy were no longer statistically significant and were deleted from the model. In the multivariate analysis (Table 5), *KRAS* mutation confirmed its association with a significantly higher risk of death (OS HR 2.17, 95% IC 1.19–3.96, *P* = 0.012). The estimate was adjusted for the statistically significant effect of adjuvant chemotherapy administration (OS HR 0.10, 95% IC 0.01–0.74, *P* = 0.024).

**Table 5:** OS multivariate analysis

Parameter	*P*‐value	OS HR	95% HR CI
*KRAS*			
Mutant versus WT	0.012	2.17	1.19–3.96
Adjuvant treatment			
Yes versus no	0.024	0.10	0.01–0.74

OS, overall survival; HR, hazard ratio; CI, confidence interval; WT, wild‐type.

## Discussion

Our molecular analysis was entirely based on metastatic resected lung tissue available from our tissue bank. The level of concordance between primary CRC and metastases in relation to *KRAS* status is known to be high, reaching a value of 94% as reported by Cejas 14. However, up to now, only two recent studies have analyzed tissue specimens from resected lung metastases 8, 12, while previous reports on *KRAS* mutational status and lung metastases considered more heterogeneous tumor sources 9, 10, 11, 13, 14, 15 (Table 6). Tie et al. evaluated oncogene mutation on liver, lung, and brain metastases from primary CRC 9, Cejas and Kim analyzed tissue samples from both primary tumor and related metastases 10, 14. In contrast, most of the studies evaluated retrospectively data of patients whose tumor was tested for *KRAS* mutation at time of diagnosis 11, 13, 15.

**Table 6:** Previous reported series of mCRC with lung metastases

Series, year	Type of samples	Number of samples	*KRAS* mutation rate (%)	*KRAS* mutation prognostic value
Cejas et al., 2009 14	Primary + various metastatic sites	110	59	Shorter DFS
Tie et al., 2011 9	Various metastatic sites	100	49	Shorter LDFS
Kim et al., 2012 10	Primary + various metastatic sites	151	45	–
Schweiger et al., 2014 8	Lung metastases	44	48	Shorter LDFS
Yaeger et al., 2015 11	Primary + various metastatic sites	918	22	Shorter OS
Pereira et al., 2015 15	Primary + various metastatic sites	494	70	Shorter LDFS
Renaud et al., 2015 12	Lung metastases	180	52	Shorter OS
Morris et al., 2014 13	Primary + various metastatic sites	484	34	Shorter OS

We found a *KRAS* mutation rate of 36%; this finding is similar to the known mutation rate in the primary tumor. Seven patients (9%) developed brain metastases.

We did not find any significant association between *KRAS* status and baseline characteristics. Univariate and multivariate analysis showed a significant association between *KRAS* wild‐type status and a better OS. Moreover, patients who did not have systemic adjuvant treatment were found to have a higher risk of death (Table 5). On the other hand, neither PFS nor LDFS were associated with *KRAS* status. A reason for this disconnection between OS, LDFS, and PFS could be given by the treatments administered before and after LM. Indeed, it must be taken into account that 9 of 48 molecularly assessed *KRAS* WT patients received an anti‐*EGFR* antibody after disease recurrence and could have had a longer OS because of these treatments. This is consistent with prior studies suggesting a predictive rather than prognostic effect of *KRAS* status 28.

Up to 75% of patients were found to have high PTEN‐negative tumors. This percentage is higher compared to that reported in other series 29, 30, 31. A possible explanation for this could be given by the heterogeneity between the different scoring systems used. Patients with intact PTEN expression had a longer survival rate compared with those whose tumors had loss of PTEN. However, the difference in survival was not statistically significant. The prognostic role of PTEN loss has not yet been clearly defined due to inconsistent results 32. In patients treated with anti‐*EGFR* antibodies, some authors reported shorter PFS and OS that reached statistical significance when this variable was combined with *PIK3CA* mutations 31. On the other hand, other authors did not find any association between PTEN protein expression and clinical outcomes 29, 30. The reason for these different results could be the small sample size of the studies, the heterogeneity of PTEN expression in primary tumor and metastatic sites and the evaluation of protein expression by IHC with different cutoff and threshold levels used for interpretation 33.

These results suggest that mCRC patients undergoing lung resection might represent a good prognosis class, in which mutant tumors for *BRAF* and *PIK3CA* in exon 20 are excluded by “natural selection”. Confirming our initial hypothesis, other studies evaluating surgery of lung metastases from CRC reported a null *BRAF* mutation rate 8, 9, while Renaud et al. reported a 10.6% *BRAF* mutation rate and identified WT *BRAF* as a positive prognostic factor for longer OS 12. *PIK3CA* mutation was not found to have any prognostic implication both in our series and in previous studies 9, 11.

Our findings do not confirm some results obtained in other series on molecular analysis on lung and central nervous system (CNS) metastases. In fact *KRAS* mutations have been previously associated with a higher CRC metastatization rate both in lung parenchyma 8, 9, 10, 11, 12, 13, 14, 15 and CNS 9, 11, and a significant association between *KRAS* mutations and relapse in the lung have also been reported 8, 9, 15. In our series, we did not assess *KRAS* status on the primary tumor. However, the observed prevalence of *KRAS* mutation is still lower than the figures reported in other studies (Table 6) and fits to the known rate of *KRAS* mutation in primary colorectal tumors. The higher rates of *KRAS* mutation reported by different authors could be due to the existing high amount of *KRAS* discordance between primary tumor and matched lung metastases, formerly reported by Kim et al. 10. In their study, the discordance rate of *KRAS* mutational status between primary and paired metastases other than the lung was 12.3%, similarly to what we have previously reported 34, while it increased in the case of lung metastases reaching a rate of 32.4% 10. We did not analyze some of the mutations comprised in the *RAS* pathway, namely *KRAS* exon 4 and *NRAS* mutations that account for up to 9% of the mutations detected in the RAS pathway 9, 35. As a matter of fact, our analysis had been performed before the data on the role of *KRAS* exon 4 and *NRAS* mutations in CRC became available.

Current National Comprehensive Cancer Network guidelines recommend follow‐up of CRC patients with chest‐abdomen‐pelvis CT scan to be performed on an annual basis 36. Nevertheless, based on the aforementioned findings, more intensive surveillance strategies have been suggested for patients with *KRAS* mutations 9, 14, 15. We cannot come to the same conclusions considering the results of our study. Indeed, among patients developing lung and brain metastases during the follow‐up, no differences were observed according to *KRAS* mutational status.

Despite the low number of patients treated with adjuvant chemotherapy (8 patients, 10% of total), this subgroup had longer PFS and OS compared to the majority (67 patients, 90%) who had no postsurgical treatment. Administration of adjuvant therapy could constitute a prognostic factor for better outcome after LM.

Although not useful in predicting recurrence pattern in mCRC, *KRAS* mutation was found to be associated with a statistically significant poorer survival rate both in univariate and multivariate analysis. *KRAS* mutation negative prognostic role has been already reported in mCRC patients undergoing hepatic metastasectomy 20, 21, 22, and there has been increasing evidence of the prognostic role of *KRAS* mutation in lung metastasectomies so far. *KRAS* may have a prognostic role in mCRC patients with lung metastases, but larger studies are needed to assess whether mutational status should be considered together with clinical and surgical parameters in the selection of patients to candidate for LM.

## Conclusions

In mCRC patients with lung metastases, we observed a significantly different pattern of metastatic spread between *KRAS* mutant and WT subgroups. In our analysis, *KRAS* mutation was associated with poorer survival in patients harboring lung metastases and might be considered having a prognostic value. Moreover, administration of adjuvant chemotherapy resulted in prolonged PFS and OS and could be considered of prognostic relevance as well.

Furthermore, larger and prospective studies are warranted to assess the possible prognostic role of *KRAS* mutational status in patients affected by mCRC and undergoing LM.

## Conflict of Interest

None declared.
